# Muscle pedicle bone flap transplantation for treating femoral neck fracture in adults: a systematic review

**DOI:** 10.1186/s13018-021-02448-9

**Published:** 2021-05-08

**Authors:** Yipeng Wu, Muguo Song, Guangliang Peng, Yongqing Xu, Yang Li, Mingjie Wei, Hui Tang, Qian Lv, Teng Wang, Xingbo Cai

**Affiliations:** Institute of Traumatology and Orthopaedics, 920th Hospital of Joint Logistics Support Force, PLA Kunming, China

**Keywords:** Muscle pedicle bone flap transplantation, Femoral neck fracture, Systematic review, Perioperative complications

## Abstract

**Background:**

This systematic review was conducted to gather available evidence on the effectiveness of muscle pedicle bone flap transplantation in adult patients with femoral neck fractures.

**Methods:**

Databases such as PubMed, EMBASE, IEEE, Web of Science, and Cochrane library were searched from their dates of inception until March 2021. Two reviewers independently selected the interventional studies on the assessment of the effectiveness of muscle pedicle bone flap transplantation for femoral neck fractures; data extraction and assessment of the methodological quality as per the Institute of Health Economics quality appraisal checklist were also performed by the reviewers. The effectiveness and complication outcomes were assessed by calculating the average rates.

**Results:**

Overall, 20 studies with 1022 patients were included in this review. Notably, the methodologic quality of the included studies was typically poor. The average effective rates were as follows: good, 73.4%; fair, 15.4%; and poor, 10.9%. Moreover, the average nonunion rate, average avascular necrosis rate, average collapse rate, and the overall reoperation rate were 9.0%, 6.7%, 4.7%, and 7.3%, respectively.

**Conclusions:**

This systematic review of heterogeneous studies with varying number of patients and varying surgical techniques indicated that muscle pedicle bone flap transplantation provides promising results with low rates of avascular necrosis and nonunion. Nevertheless, further controlled studies are required to ascertain the effectiveness of muscle pedicle bone flap transplantation in treating femoral neck fracture.

## Background

Hip fracture is associated with limited movement, chronic pain, disability, loss of independence, and decline in the quality of life. Moreover, approximately 20–30% of patients with hip fractures die within a year [1, 2]. Notably, femoral neck fracture is the most common type of hip fracture. Femoral neck fracture treatment is typically classified into conservative and surgical treatments. Nevertheless, because conservative treatment requires long-term bedrest, the incidence of complications such as pulmonary infection and thrombosis is high. Therefore, most clinicians recommend surgical treatment as the first-line of treatment in old patients with femoral neck fracture [3]. Surgical treatment of displaced intracapsular neck fractures in patients aged more than 70 years entails hip replacement with a partial or total prosthesis—a modality accepted by a vast majority of researchers worldwide. Nonetheless, the conservative treatment is typically recommended in younger patients (< 60 years). Moreover, complications such as fracture nonunion and osteonecrosis of the femoral head can easily occur after the femoral neck fracture [4]. The current, tried, and tested surgical treatment methods for ununited femoral neck fractures are internal fixation, internal fixation plus osteotomy with or without bone graft, non-vascularized or vascularized bone graft, and hip arthroplasty [5]. Nevertheless, in developing countries, various factors such as illiteracy, low socioeconomic status, ignorance, and poor medical facilities might cause a delay in surgical treatment. Squatting and sitting cross-legged are inherently involved in the activities of daily living, particularly in a developing country such as India. Therefore, considering the needs of such patients, as well as the cost of joint replacement surgeries, salvaging the femoral head is of paramount importance, and several patients opt for femoral head salvage surgery.

The surgical treatment of femoral neck fracture remains controversial despite several advancements in the orthopedics domain. Nonunion and avascular necrosis are the two major complications of this fracture. Although the rate of nonunion has been reduced through anatomical reduction and stable fixation of fractures, the incidence of avascular necrosis is high [6]. In 1962, an autogenous muscle pedicle graft from the quadratus femoris muscle was used for the first time [7]. In addition, the application of fresh autogenous cancellous iliac bone chips along with muscle pedicle bone grafting has been reported to provide excellent outcomes [8]. However, whether muscle pedicle bone flap transplantation is effective in treating femoral neck fractures remains inconclusive.

It is very important to determine the effectiveness of muscle pedicle bone flap transplantation and its potential related factors. This not only builds a bridge between clinical and basic or translational science, but also for complex surgical problems, it is necessary for clinicians to understand the disease process, integrate new concepts into their surgical techniques, integrate new scientific discoveries, and improve the operation practice in the operating room [9, 10].

Therefore, this systematic review analyzed the available evidence on the efficacy and safety of muscle pedicle bone flap transplantation for femoral neck fractures in adults.

## Methods

A systematic review was performed in accordance with the Cochrane Systematic Review Guidelines and the Preferred Reporting Items for Systematic Reviews and Meta-Analyses checklist [11, 12]. This systematic review is based on the literature. All previously published studies were analyzed, and thus, ethical approval and patient consent were not required.

### Search strategy

Databases including PubMed, EMBASE, IEEE, Web of Science, and Cochrane library were searched from their date of inception until March 2021 to identify studies that have assessed the efficacy of muscle pedicle bone flap transplantation in treating femoral neck fracture. No language restrictions were applied. The following search terms were used: “femoral neck fracture,” “fracture of femoral neck,” “muscle pedicle,” “bone flap,” and “bone grafting.” In addition, the references of all the retrieved articles, including the relevant systematic reviews, were manually searched for additional relevant articles.

### Study selection criteria

The inclusion and exclusion criteria for population, intervention, comparison, outcomes, and study were defined and applied.

#### Participants

Participants include adult patients diagnosed with femoral neck fractures, including displaced femoral neck fracture, ununited femoral neck fracture, and neglected femoral neck fracture. However, trials focusing on the treatment of patients with fracture not limited to femoral neck as well as reoperation were excluded.

#### Intervention and comparison

This review included studies on any type of muscle pedicle bone flap transplantation involving tensor fascia lata muscle, gluteus medius muscle, quadratus femoris muscle, and sartorius muscle pedicle bone grafting. However, studies that focused on bone grafting without muscle pedicle were excluded.

#### Outcomes

Primary outcomes were nonunion and avascular necrosis rates, whereas the secondary outcomes were the collapse rate, reoperation rate, and effective rate. Studies that did not report the eligible outcomes or data were excluded.

#### Study

Published or unpublished randomized controlled trials (RCTs) or non-RCTs were selected. In addition, case series were included. However, reviews or animal experiments were excluded.

The selection of studies was conducted independently by two reviewers. After removing duplicates, the reviewers screened the titles and abstracts of all the identified studies. Full text of all articles with potential relevance were retrieved for comprehensive assessment as per the inclusion criteria. Any disagreement was resolved through consensus with a third reviewer.

### Data collection and analysis

All study characteristics and data, such as study population, sample size, and outcomes, were extracted as per the predefined criteria. Two authors independently extracted the data using a data extraction form. Any potential disagreement between the authors was resolved, and consensus was established through discussion involving a third author.

### Quality assessment

In addition, two authors independently evaluated the methodological quality. The case series was assessed on the basis of the Institute of Health Economics quality appraisal checklist form [13]. This 20-criterion checklist has eight aspects, namely, study objective, study design, study population, intervention and co-intervention, outcome measures, statistical analysis, results, and conclusions, as well as competing interests and sources of support. Notably, criteria such as prospective study, consecutive recruitment, predefined inclusion or exclusion criteria, before and after outcome measurement, and sufficient follow-up data were used to examine how the study was executed, whereas other criteria (such as a clear statement of study objective, description of patient characteristics, interventions and co-interventions, reporting of adverse events, competing interests, and sources of support) focused on the reporting quality. The items were rated as follows: yes, unclear or partial, and no. Any discrepancies were resolved through discussion between all the authors.

### Statistical analysis

Notably, both meta-analytical and level of evidence approaches were deemed inappropriate to formulate conclusions because of the inadequacy of comparison. The average nonunion, avascular necrosis, collapse, reoperation, and effective rates were calculated on the basis of the sum of the number of patients who experienced these events divided by the sum of the number of patients who received muscle pedicle bone flap transplantation.

## Results

**Fig. 1:**
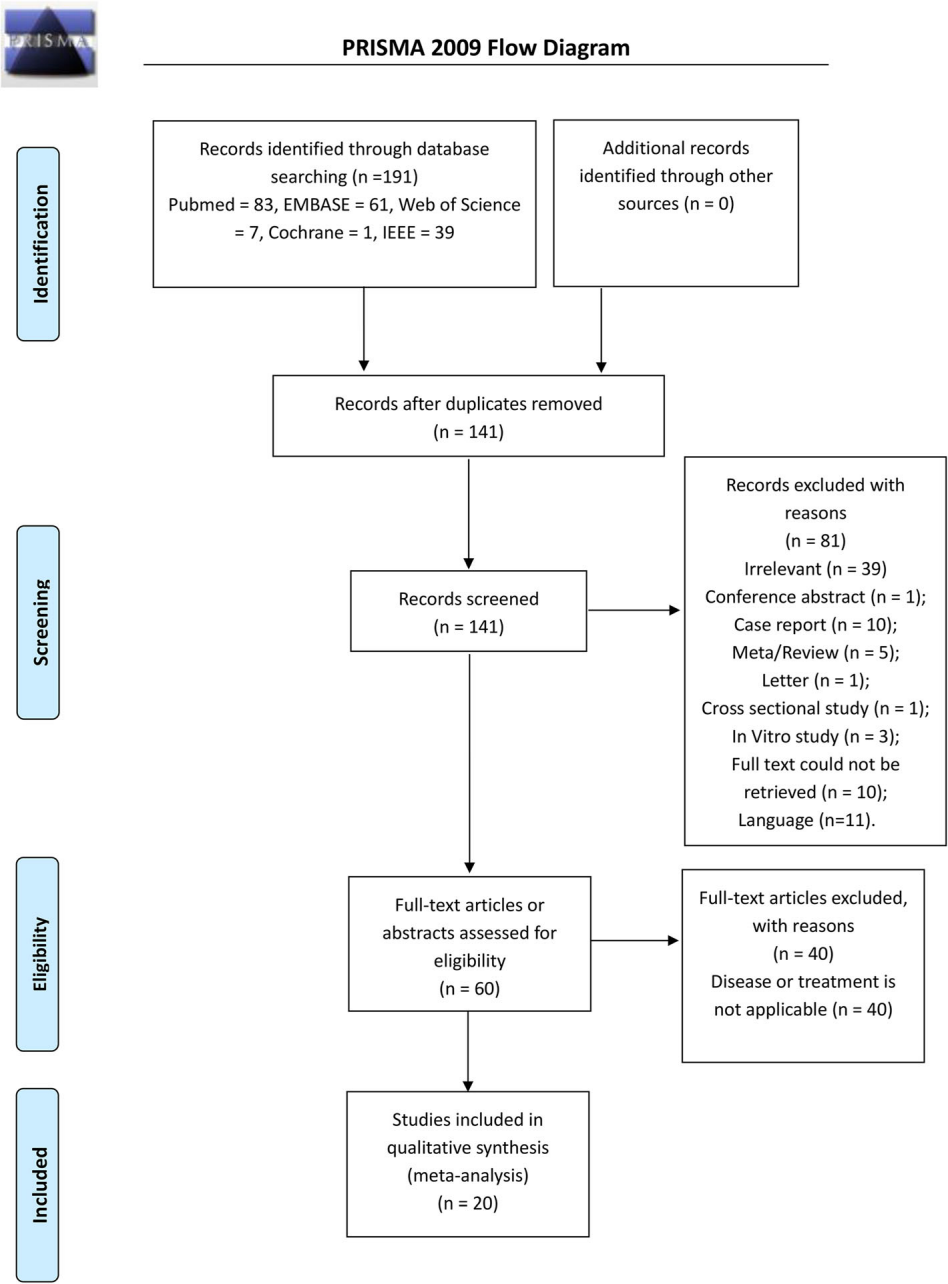
Summary of the identification and selection process of relevant literature

After the initial database search and removal of duplicates, provided a total of 147 potentially relevant articles, of which 45 duplicate publications were excluded. Of the remaining 102 articles, 42 were excluded after screening of the title and abstract. The remaining 60 articles, which included 40 studies with unapplicable disease or treatment, were excluded. Finally, 20 trials were included in the review [8, 14–32]. Figure 1 illustrates the selection process of the studies.

### Study characteristics

All the included studies were published between 1973 and 2020. Of the included studies, four were retrospective in nature; two, case-control studies; one, a non-randomized control trial; and the remaining were case series. No RCT was included in this systematic review. Of the 20 publications, 3 were from the USA, 5 were from China, 1 was from Turkey, and the remaining were from India. The procedures used in these studies included quadratus femoris muscle pedicle bone grafting with screw fixation, open reduction and internal fixation, tensor fascia lata, and gluteus medius muscle pedicle bone grafting. Moreover, the included studies focused on patients with femoral neck fractures, mainly displaced fracture of the femoral neck, ununited femoral neck fractures, transcervical or subcapital fractures of the femoral neck, and neglected femoral neck fracture. Table 1 lists the characteristics of the studies included in the present meta-analysis.

**Table 1:** Characteristics of the studies included in the systematic review

ID	Region	Study design	Age (years, mean, or range)	Male (%)	Patients	Complications	Cause	Fracture classification	Sample size	Intervention	Follow-up (months)	Outcomes
Meyers, 1973 [8]	USA	Retrospective study	36 (24%) of the patients were below the age of 55	43.3	Displaced subcapital and transcervical femoral neck fractures	Alcoholism (delirium tremens and severe liver damage)	Alcoholics	Subcapital and transcervical fractures	150	Muscle-pedicle-bone graft and internal fixation	3–48	Nonunion rate, collapse rate
Meyers, 1975 [32]	USA	Case series	21–39	73.9	Displaced fracture of the femoral neck	NA	Severe trauma	Concomitant ipsilateral fracture of the femur (femoral shaft or intertrochanteric area)	23	Open reduction and internal fixation as well as muscle-pedicle graft	13 patients were followed for over 18 months and 9, for over 24 months	Nonunion rate
Morwessel, 1985 [31]	USA	Retrospective study	39 (20–60)	76.9	Displaced femoral neck fracture	Other associated injuries	Fall and motor vehicle accident	Subcapital fractures (Garden type III or IV)	13	Quadratus femoris muscle pedicle bone grafting with screw fixation	38 (17–100)	Nonunion rate, collapse rate, reoperation rate
Baksi, 1986 [30]	India	Case series	42 (11–67)	33.9	Ununited femoral neck fractures	Renal diabetes, hypertension, psychiatric disorders	NA	Intracapsular fracture	56	Internal fixation combined with muscle-pedicle bone grafting	34.6 (18–82)	Nonunion rate
Biswas, 1997 [29]	India	Case series	18–47	100	Non-union of fracture neck femur of 6–12 months	NA	Road traffic accident, fall from bicycle and falls on uneven ground	Intracapsular fracture	12	Open reduction and internal fixation and tensor fascia lata or gluteus medius muscle pedicle bone grafting	18 (6–24)	Nonunion rate, effective rate
Liu, 2003 [28]	China	Case-controlled	42.5/43.5	30/30.4	Fresh transcervical or subcapital fractures of the femoral neck	NA	NA	Subcapital fractures or transcervical	30/23	Muscle bone flap of both sartorius with or without tensor fasciae Iatae	48 (36–60)	Reoperation rate, effective rate
Yang, 2006 [27]	China	Case series	30.6 (21–49)	60.5	Femoral neck fractures	NA	Traffic accident, falls from height, and other falls	Garden type III or IV	86	Compressed screw and sartorius bone flap	29 (6–84)	Nonunion rate, avascular necrosis rate, reoperation rate, effective rate (Kan Wusheng)
Gupta, 2007 [26]	India	Case series	24 (10–40)	80	Ununited fractures of the femoral neck	ununited fractures	NA	Intracapsular fracture	20	Internal fixation and muscle pedicle periosteal grafting	70 (14–144)	nonunion rate
Chaudhuri, 2008 [25]	India	Case series	54.6 (24–81)	63	Fresh displaced femoral neck fracture	NA	Road traffic accident and falls at their residence	Garden stage III and stage IV	73	Quadratus femoris muscle pedicle bone grafting	67.2 (24–132)	Nonunion rate, Reoperation rate, coxa vara rate, avascular necrosis rate, effective rate
Gupta, 2008 [24]	India	Case series	45 (14–62)	81.3	Displaced femoral neck fractures	NA	Road traffic accident, falls from height, and slip while walking.	Garden stage III, IV	32	Open reduction and internal fixation with muscle pedicle grafting	40.8 (24–102)	Avascular necrosis rate, coxa vara rate, transient foot drop rate, temporary loss of scrotal sensation rate
Yun, 2010 [22]	China	Case series	40 (25–60)	57.9	Femoral neck fractures	NA	Traffic accident, fall from height, and combined injury	Garden stage II, III	38	Quadratus femoris muscle pedicle bone graft with screw fixation	24–60	Nonunion rate, avascular necrosis rate, effective rate
Vallamshetla, 2010 [23]	India	Retrospective study	34 (24–51)	66.7	Ununited intracapsular femoral neck fractures	NA	NA	NA	42 (14 females)	Quadratus femoris muscle pedicle bone grafting	63 (36–84) (range, 3–7 years)	Nonunion rate, failures rate, infection rate, varus union rate, avascular necrosis rate
Bhuyan, 2012 [21]	India	Case series	32.9 (20–53)	66.7	Neglected intracapsular femoral neck fracture	Associated injuries	Road traffic accident, fall from height	NA	48	Tensor fascia latae muscle pedicle bone grafting	52.8 (24–81.6)	Nonunion rate, avascular necrosis rate, coxa vara rate, effective rate
Zha, 2014 [18]	China	Non-randomized control	41.98 ± 6.8	70	Garden III/IV femoral neck fracture	NA	NA	Garden stage III, IV	30	Quadratus femoris muscle pedicle bone flap transplantation combined with hollow compression screw fixation	6	Nonunion rate, avascular necrosis rate, collapse rate, effective rate
Mishra, 2014 [20]	India	Retrospective study	38 (15–51)	66.7	Neglected femoral neck fracture	Associated injuries	Road traffic accident, slip on the ground while walking and fall from the height	Garden stage III, IV	36	Triple muscle (sartorius, tensor fascia latae, and part of gluteus medius) pedicle bone grafting	54 (24–168)	Nonunion rate, coxa vara rate, avascular necrosis rate, effective rate
Nair, 2014 [19]	India	Case series	26.69 (15–45)	82.4	Neglected and ununited femoral neck fracture	Neck resorption	Road traffic accident, fall from height	NA	17	Quadratus femoris muscle pedicle bone grafting along with open reduction and internal fixation (ORIF)	NA	Shortening rate, coxa vara rate, effective rate (PMA scoring)
Zhang, 2015 [17]	China	Case-control study	37.8 ± 6.9	76.9	Garden III/IV femoral neck fracture	NA	Traffic accident, fall from height and other	Garden stage III, IV	26	Quadratus femoris bone flap transplantation	32 (28–41)	Nonunion rate, Harris hip score, avascular necrosis rate
Baksi, 2016 [16]	India	Case series	43.3 (16–55)	54.1	Ununited femoral neck fracture	NA	NA	NA	244	Internal fixation combined with iliac crest bone chips and muscle pedicle bone grafting	150 (36–420)	Nonunion rate, coxa vara rate, Harris hip score, hip motions, avascular necrosis rate
Salgotra, 2016 [15]	India	Case series	47 (38–55)	85.7	Delayed femoral neck fractures	NA	NA	Subcapital fractures or transcervical	7	Muscle-pedicle bone grafting with tensor fascia lata	36	Nonunion rate, collapse rate, total hip replacement rate, modified Harris hip score
Tuzun, 2020 [14]	Turkey	Case series	36.3 (19–58)	56.3	Ununited femoral neck fractures	Atrophy	Traffic accident, fall from height	NA	16	Quadratus femoris muscle pedicle bone grafting	24	Avascular necrosis rate, reoperation rate, Harris hip scores

### Quality assessment

Table 2 presents the methodological quality of the studies. Losses to follow-up, adverse events, and the conclusions supported by the results were reported in all but one study [14]. The objective was not clearly stated in one study [30]. Except for four studies, all the studies were conducted prospectively [18, 21, 27, 31]. Only three studies recruited consecutive patients [14, 23, 25]. Regarding intervention and co-intervention, except for two studies, all the studies clearly described the intervention of interest [15, 19]. All studies except three [15, 19, 32] had clearly described the additional interventions. Regarding the outcome measures, no study had reported the statistical tests used to appropriately assess the relevant outcomes, except for two, which provided estimates of random variability in the data analysis of relevant outcomes [14, 15]. Overall, the methodological quality of the included studies was generally poor.

**Table 2:** Methodologic quality of the case-series

Item	Meyers, 1975 [32]	Morwesselv, 1985 [31]	Biswas, 1997 [29]	Yang, 2006 [27]	Gupta, 2007 [26]	Chaudhuri, 2008 [25]	Gupta, 2008 [24]	Yun, 2010 [22]	Bhuyan, 2012 [21]	Nair, 2014 [19]	Baksi, 2016 [16]	Salgotra, 2016 [15]	Tuzun, 2020 [14]
**Study objective**													
1. Was the hypothesis/aim/objective of the study clearly stated?	Yes	Yes	Yes	Yes	Yes	Yes	Yes	Yes	Yes	Yes	No	Yes	Yes
**Study design**													
2. Was the study conducted prospectively?	Yes	No	Yes	No	Yes	Yes	Yes	Yes	No	Yes	Yes	Yes	Yes
3. Were the cases collected in more than one center?	No	No	Unclear	No	Unclear	Unclear	Unclear	No	Yes	No	Unclear	Unclear	Unclear
4. Were patients recruited consecutively?	Unclear	Unclear	Unclear	Unclear	Unclear	Yes	Unclear	No	Yes	Unclear	Unclear	Unclear	Yes
**Study population**													
5. Were the characteristics of the patients included in the study described?	Partial	Partial	Yes	Yes	Yes	Yes	Yes	Yes	Yes	Yes	Yes	Partial	Yes
6. Were the eligibility criteria (i.e., inclusion and exclusion criteria) for entry into the study clearly stated?	Partial	Partial	Yes	No	No	Yes	Yes	Yes	Yes	Yes	Yes	Partial	No
7. Did patients enter the study at a similar point in the disease?	No	No	Yes	No	No	Yes	Yes	No	No	Yes	Yes	No	Yes
**Intervention and cointervention**													
8. Was the intervention of interest clearly described?	Yes	Yes	Yes	Yes	Yes	Yes	Yes	Yes	Yes	Partial	Yes	Partial	Yes
9. Were additional interventions (cointerventions) clearly described?	No	Yes	Yes	Yes	Yes	Yes	Yes	Yes	Yes	No	Yes	Partial	Yes
**Outcome measures**													
10. Were relevant outcome measures established as priority?	No	No	Yes	Yes	No	No	No	Yes	Yes	Yes	No	No	Yes
11. Were outcome assessors blinded to the intervention that patients received?	No	No	No	No	No	No	No	No	No	No	No	No	No
12. Were the relevant outcomes measured using appropriate objective/subjective methods?	Partial	Partial	Partial	Yes	Partial	Yes	Yes	Yes	Yes	Yes	Yes	Yes	Yes
13. Were the relevant outcome measures made before and after the intervention?	Yes	Yes	Yes	No	Unclear	Yes	Yes	Yes	Yes	Yes	Yes	Yes	Yes
**Statistical analysis**													
14. Were the statistical tests used to assess the relevant outcomes appropriate?	Unclear	Unclear	Unclear	Unclear	Unclear	Unclear	Unclear	Yes	Unclear	Unclear	Unclear	Unclear	Yes
**Results and conclusions**													
15. Was follow-up long enough for important events and outcomes to occur?	Yes	Yes	Yes	Yes	Yes	Yes	Yes	Yes	Yes	Unclear	Yes	Yes	Yes
16. Were losses to follow-up reported?	Yes	Yes	Yes	Yes	Yes	Yes	Yes	Yes	Yes	Yes	Yes	Yes	No
17. Did the study provided estimates of random variability in the data analysis of relevant outcomes?	No	No	No	No	Partial	No	No	No	No	Partial	Partial	Yes	No
18. Were the adverse events reported?	Yes	Yes	Yes	Yes	Yes	Yes	Yes	Yes	Yes	Yes	Yes	Yes	Yes
19. Were the conclusions of the study supported by the results?	Yes	Yes	Yes	Yes	Yes	Yes	Yes	Yes	Yes	Yes	Yes	Yes	Yes
**Competing interests and sources of support**													
20. Were both competing interests and sources of support for the study reported?	Partial	Partial	Partial	No	Yes	Yes	Yes	No	Yes	Yes	Yes	Yes	Yes

### Complications

Among the included studies, 17 studies with 877 patients reported an average nonunion rate of 9.0% (95% CI, 7.2–11.0%). The average avascular necrosis rate of 6.7% was reported by 11 studies that enrolled 644 patients (95% CI, 3.6–10.8%).

The collapse rate was reported by 6 studies with 471 patients, and its 95% CI ranged from 3.0 to 6.8% (average, 4.7%). The reoperation rate was reported by eight studies that enrolled 546 patients, and its 95%CI ranged from 2.9 to 13.4% (average, 7.3%). The summary of complications is listed in Table 3.

**Table 3:** Summary of complications and efficacy outcomes in the included studies

	*N*	Rate (95% CI)	*I*^2^	*P* (heterogeneity)
Nonunion rate	17	0.090 (0.072, 0.110)	0	0.520
Collapse rate	6	0.047 (0.030, 0.068)	30.73	0.205
Effective rate				
Poor	10	0.109 (0.072, 0.154)	55.26	0.017
Fair	10	0.154 (0.088, 0.234)	81.81	< 0.001
Good	10	0.734 (0.626, 0.830)	86.33	< 0.001
Reoperation rate	8	0.073 (0.029, 0.134)	78.67	< 0.001
Avascular necrosis rate	11	0.067 (0.036, 0.108)	65.41	0.001
Coxa vara rate	6	0.101 (0.051, 0.166)	67.95	0.008

### Effectiveness outcome

The effective rate was reported by 10 studies involving 612 patients; of these, three used self-established criteria [27–29], four used the modified Harris hip score [16, 18, 21, 25], one used the Salvati and Wilson score [20], one study used the Sanders score [22], and one study used the modified Postel and Merle d’Aubigne hip scoring to assess the effective rate [19]. An average effective rate of 73.4% were considered good, while rates of 15.4% and 10.9% were considered fair and poor, respectively. Table 3 presents the summary of the efficacy outcome.

## Discussion

### Summary of evidence

We identified 20 studies that met our inclusion criteria. These studies included 1022 adult patients with femoral neck fractures who were treated with muscle pedicle bone flap transplantation. Notably, our systematic review indicated an average good effective rate of 73.4% for the muscle pedicle bone flap transplantation. Furthermore, the nonunion rate of muscle pedicle bone flap transplantation was 9.0%, the avascular necrosis rate was 6.7%, the collapse rate was 4.7%, and the reoperation rate was 7.3%.

One case series and literature review had summarized the evidence of muscle pedicle bone flap transplantation application in treating femoral neck fractures in adults [15]. However, the review included only six studies, and no quality assessment was conducted in the review. In that review, the muscle pedicle bone flap was compared with other treatments for delayed or ununited fractures of the femoral neck, and it claimed that the vascularized iliac crest, fibular, and periosteal grafting procedures are not popular procedures due to their time-consuming and technically demanding nature as well as due to the need for high competency from average orthopedic surgeons. Furthermore, despite the high union rate of such vascularized bone grafting series, these reviews were small and had short follow-up durations; therefore, it is challenging to predict the future occurrence of avascular necrosis in such patients. Conversely, the follow-up duration in the studies included in our systematic review was longer; the duration was approximately 3 years in 10 studies, and considerably longer in other studies (e.g., the average study duration in one study was 150 months), which is sufficiently long to predict the future occurrence of avascular necrosis rate in those patients.

### Nonunion and avascular necrosis rates of muscle pedicle bone flap transplantation

Femoral head viability remains a major concern in femoral neck fractures. The two most challenging complications of femoral neck fractures in young adults are femoral head osteonecrosis and nonunion. Notably, osteonecrosis is a devastating complication in young patients due to the limited availability of options for young patients compared with that for elderly patients with the same condition afflicting the femoral head [5]. Despite advancements in surgical techniques, instrumentations, and imaging modalities, complications such as nonunion (10–30%) and avascular necrosis (15–33%) persist in affected patients [33, 34]. The average nonunion and avascular necrosis rates of muscle pedicle bone flap transplantation in our systematic review of 14 studies were less than 10%. Moreover, bone grafting has evolved as a treatment modality for these fractures with predictable results in the long term. Furthermore, if used along with internal fixation in neglected femoral neck fracture, the vascularized bone grafting on a muscle pedicle, such as gluteus medius, quadratus femoris, or sartorius, further supplies blood to the femoral head by acting as a vascular inlay graft and structural bone graft to buttress the posterior femoral neck comminution and enhances stability, thereby improving osteosynthesis [35]. Nevertheless, risk factor analysis for nonunion and avascular necrosis rates was not performed in these studies; therefore, the systematic review could not provide suggestions to doctors regarding the nonunion and avascular necrosis rates.

Nonetheless, the muscle pedicle bone grafting approach is associated with the risk of extensive dissection and blood loss, particularly the risk of injuring the medial femoral circumflex artery. Commonly encountered problems with this approach are the need for experienced surgeons with excellent technical skills, long procedural duration, extensive soft tissue dissection, blood loss, and high risk of postoperative shock and infection [8]. Moreover, any torsion or tension in the muscle pedicle must be avoided when transferring the muscle pedicle bone graft to its recipient site [36]. The average duration of anesthesia and surgery in patients was 3.44 h. Time variations could be attributed to differences in the skill and experience of the surgeon.

### Limitations of this review

Our literature search was comprehensive without any language restrictions; however, we cannot rule out the availability of other small and unpublished trials. Moreover, the evidence in this review is limited because of the small sample sizes and low methodologic quality of the included studies. Notably, half of these studies had fewer than 20 patients, and the individual differences in rates were large. For example, Hou et al. reported a collapse rate of 0.00% in 5 patients, whereas Morwessel et al. reported a collapse rate of 23.08% in 13 patients [29, 34]. Furthermore, most of the studies did not report the details pertaining to the diagnosis of the collapse and nonunion rates; thus, the consistency in these rates across the included studies could not be guaranteed. In addition, more than half of the studies (11 of 20) were from India, which may induce a risk of bias.

Moreover, there was no comparison of these case series, and thus, no meta-analysis could be conducted. Because most of the case series did not assess hip function by using functional parameters, such as the modified Harris hip score or Postel and Merle d’Aubigne’s hip scoring, this review could not evaluate the functional recovery.

Based on the results of the present analysis of 20 articles, we found that the average effective rate of muscle pedicle bone flap transplantation was 73.4%, with a nonunion rate of 9.0%, avascular necrosis rate of 6.7%, collapse rate of 4.7%, and reoperation rate of 7.3%. Our study has relatively low rates of nonunion and avascular necrosis, and if performed along with internal fixation and grafting of the vascularized bone on a muscle pedicle, it can provide further blood supply to the femoral head, enhance stability, and improve blood supply to the femoral head. However, risk factor analyses for nonunion and avascular necrosis rates were not performed in these studies; therefore, the systematic review could not provide suggestions to doctors regarding the nonunion and avascular necrosis rates.

## Conclusions

Our systematic review of heterogenous studies with varying numbers of patients and varying surgical techniques indicated that muscle pedicle bone flap transplantation provides promising results with low rates of avascular necrosis and nonunion. Nonetheless, further research is needed to confirm the efficacy of muscle pedicle bone flap transplantation in treating fracture of the femoral neck.

## Data Availability

The datasets used and/or analyzed during the current study are available from the corresponding author on reasonable request.

## Abbreviations

IHE: Institute of Health Economics
PICOS: Population, intervention, comparison, outcomes, and study
RCTs: Randomized controlled trials
